# Assessing the synergy between cholinomimetics and memantine as augmentation therapy in cognitive impairment in schizophrenia. A virtual human patient trial using quantitative systems pharmacology

**DOI:** 10.3389/fphar.2015.00198

**Published:** 2015-09-22

**Authors:** Hugo Geerts, Patrick Roberts, Athan Spiros

**Affiliations:** ^1^In Silico BiosciencesBerwyn, PA, USA; ^2^Perelman School of Medicine, University of PennsylvaniaPhiladelphia, PA, USA; ^3^Department of Veterinary and Comparative Anatomy, Pharmacology and Physiology, Washington State UniversityPullman, WA, USA

**Keywords:** cognition, polypharmacy, antipsychotics, cholinomimetic, schizophrenia

## Abstract

While many drug discovery research programs aim to develop highly selective clinical candidates, their clinical success is limited because of the complex non-linear interactions of human brain neuronal circuits. Therefore, a rational approach for identifying appropriate synergistic multipharmacology and validating optimal target combinations is desperately needed. A mechanism-based Quantitative Systems Pharmacology (QSP) computer-based modeling platform that combines biophysically realistic preclinical neurophysiology and neuropharmacology with clinical information is a possible solution. This paper reports the application of such a model for Cognitive Impairment In Schizophrenia (CIAS), where the cholinomimetics galantamine and donepezil are combined with memantine and with different antipsychotics and smoking in a virtual human patient experiment. The results suggest that cholinomimetics added to antipsychotics have a modest effect on cognition in CIAS in non-smoking patients with haloperidol and risperidone and to a lesser extent with olanzapine and aripiprazole. Smoking reduces the effect of cholinomimetics with aripiprazole and olanzapine, but enhances the effect in haloperidol and risperidone. Adding memantine to antipsychotics improves cognition except with quetiapine, an effect enhanced with smoking. Combining cholinomimetics, antipsychotics and memantine in general shows an additive effect, except for a negative interaction with aripiprazole and quetiapine and a synergistic effect with olanzapine and haloperidol in non-smokers and haloperidol in smokers. The complex interaction of cholinomimetics with memantine, antipsychotics and smoking can be quantitatively studied using mechanism-based advanced computer modeling. QSP modeling of virtual human patients can possibly generate useful insights on the non-linear interactions of multipharmacology drugs and support complex CNS R&D projects in cognition in search of synergistic polypharmacy.

## Introduction

While polypharmacy is more of a rule than an exception in real-life clinical treatment, preclinical animal models are ill-equipped to address the issue of comedication because of fundamental species-specific differences in drug metabolism, the impact of human-specific genotypes, the different pharmacology of the drugs for human vs. rodent targets and incomplete pathology (for a review see, Geerts, 2009).

Cognitive Impairment in Schizophrenia (CIAS) is a major unmet medical need for this patient population; while psychosis can be readily managed by the current antipsychotic drug armentarium, cognitive and negative symptoms are hampering patients to return to a more normal professional life (Kitchen et al., 2012). This has prompted the major stakeholders from industry, regulatory agencies and academia to develop a regulatory path for cognitive enhancement, resulting in the development of the Matrics battery (Green and Nuechterlein, 2004). Over the last 15 years, many novel highly selective drugs have been tested for cognitive enhancement as augmentation therapy without much success (Dunlop and Brandon, 2015).

Symptomatic treatment has been successful in Alzheimer's disease with cholinomimetics and memantine. Galantamine is an acetylcholinesterase inhibitor (AChE-I) with allosteric potentiating effects on nicotinic receptors (nAChR) currently approved for Alzheimer's Disease (Tariot et al., 2000) and has been tested for cognitive improvement in schizophrenia (Norén et al., 2006; Deutsch et al., 2008; Dyer et al., 2008; Sacco et al., 2008). Donepezil, a pure AChE-I has been studied for cognitive improvement in schizophrenia with mixed results (Akhondzadeh et al., 2008; Keefe et al., 2008; Gauthier and Molinuevo, 2013). On the other hand, memantine is a weak NMDA-antagonist approved for moderate-to-severe AD (Reisberg et al., 2003) with preferential affinity against the excitatory-inhibitory synapses in cortical networks (Kotermanski and Johnson, 2009). The effect of these compounds in CIAS has yielded equivocal results. Possible reasons for the lack of clear results include non-trivial pharmacodynamic relationships between the investigative drugs and the different baseline antipsychotics. For instance, some antipsychotics such as olanzapine and clozapine affect the muscarinic cholinergic receptors; indeed olanzapine is a documented antagonist of M_1_, M_2_, M_3_, M_4_, and M_5_ mAChR with respective affinities of 26, 18, 52, 17, and 20 nM (Bymaster et al., 1996). Therefore, it is to be expected that the dose-response of cholinomimetics in CIAS might be highly dependent upon the different antipsychotics.

To complicate the situation even further, anticholinergics are often used in treating the symptoms of extrapyramidal motor symptoms and can affect the cognitive function substantially (Ogino et al., 2014). In addition, a disproportionate fraction of schizophrenia patients tend to smoke (Dalack et al., 1998), adding more complexity to the pharmacodynamic interaction of cholinomimetics at the level of nicotinic receptors.

A recent paper suggested that the combination of memantine and AChE inhibitors, in particular galantamine, would show a bigger effect in CIAS because of their complementary pharmacology on pyramidal cells and interneurons (Koola et al., 2014).

Testing such a combination therapy is likely beyond the capability of preclinical animal testing due to the complexity and combinatorial challenges of the trial design. To explore the possible clinical applications of this combination therapy, other approaches need to be explored. In this paper we propose an advanced version of a computer-based Quantitative Systems Pharmacology (QSP) platform, a mechanism-based computer model of the relevant humanized cortical networks that has been developed for clinical readouts in psychiatry and neurology, and calibrated with group average clinical data. Such an approach is similar to Physiology-Based PharmacoDynamic (PBPD) Modeling in line with new terminology around Physiology-Based Pharmacokinetic Modeling (PBPK) and is gaining traction in pharmaceutical research and development. The platform has been able to blindly, prospectively and correctly predict an unexpected clinical outcome in schizophrenia and Alzheimer's disease (AD) (Geerts et al., 2012; Nicholas et al., 2013; Liu et al., 2014) and has been calibrated for clinical cognitive outcomes in conditions of chronic schizophrenia (Geerts et al., 2013).

Testing the platform in a number of practical clinical situations with known outcomes, such as the augmentation therapy of cholinomimetics and memantine on antipsychotics is mandatory to help constrain the platform. Once calibrated and constrained, with human clinical data, this QSP approach can then be used in rationally designed multi-target drug discovery programs.

## Methods

We use a previously described (Geerts et al., 2013) biophysically realistic and mechanism-based QSP platform to simulate the impact of augmentation therapy with cognitive enhancers in virtual schizophrenia patients. This QSP platform has been calibrated against observed clinical effects on the N-back working memory test with various therapeutic interventions in diverse patient populations and recapitulates the negative pharmacodynamic clinical effect of risperidone augmentation on clozapine. Basically, the platform consists of a receptor competition model that allows accurate quantification of drug target exposure, a biophysically realistic neuronal network that captures the microarchitecture of a cortical column and a calibration module that relates computer model outcome to actual clinical results. The platform includes the neurophysiology of over 30 CNS targets, ranging from catecholamine GPCR over various glutamate, GABA receptors and ligand and voltage-gated ion channels to enzymes such as Catechol-O-methyl transferase (COMT) and PDE10 and neurotransmitter transporters.

### Defining target exposure in quantitative systems pharmacology model

The receptor competition model (Spiros et al., 2010; Spiros and Geerts, 2012) calculates the degree of activation of various postsynaptic receptors (dopamine, serotonin, norepinephrine, and cholinergic neurotransmitters) in the presence of antipsychotics. The affinity of the parent molecule and its major metabolite for both pre- and post-synaptic receptors, derived from the Psychoactive Drug Screening Program (Besnard et al., 2012) is used to calculate the competition with endogenous neurotransmitters. The presynaptic autoreceptor neurophysiology properties are calculated from preclinical data using fast-cyclic voltammetry constrained with clinical imaging data (Nicholas et al., 2013). The functional intrasynaptic concentration of the specific antipsychotic is determined from calculating the concentration that corresponds to the clinically observed displacement of a radio-active D_2_R specific PET tracer, such as raclopride (Spiros et al., 2012). We assume regular clinical doses 400 and 600 mg, i.e., 6 mg risperidone, 10 mg haloperidol, 15 mg olanzapine, 200 quetiapine, and 30 mg aripiprazole.

### Quantitative systems pharmacology model for cognitive impairment in schizophrenia

The QSP model consists of a network of 80 four-compartment pyramidal and 40 two-compartment interneurons with the effects of dopaminergic, serotonergic, noradrenergic and cholinergic modulation (including a spatio-temporal receptor state model for allosteric modulation of the different nicotinic ACh receptors) and has been described in detail elsewhere (Geerts et al., 2013). A subset of pyramidal cells is stimulated for a very short period at a certain time point, reflecting a sensory stimulus typical of a working memory paradigm. The actual membrane potential of each compartment can be calculated from the actual conductances, which are dependent upon the activation level of various G-protein coupled receptors. The resulting state diagram shows a synchronized firing of the cells during 5–10 s after they have been stimulated for a short period (100 ms). While this computational neuroscience model has been designed using *in vivo* electrophysiological single-unit recordings in non-human primates (Williams and Goldman-Rakic, 1995) performing a working memory task and therefore probably only reflects the maintenance phase, the outcome could be generalized to the strength of a memory trace (Roberts et al., 2012; Geerts et al., 2013). We have shown previously that the duration of this synchronized firing correlates well with actual 2-Back working memory task in a variety of experimental interventions in humans (Geerts et al., 2013).

Schizophrenia pathology is implemented using insights from human neuroimaging, genetic and neuropathology data and includes a hypodopaminergic cortical D_1_R tone (Durstewitz and Seamans, 2008), NMDA-R hypofunction (Coyle, 2006) documented by a hypocortical-hyperstriatal imbalance in metabolic imaging (Meyer-Lindenberg et al., 2002), a GABA deficit (Volk and Lewis, 2002) applied here to the network interneurons, and a noisier background signal (Winterer et al., 2000), resulting in a clinical cognitive deficit which is dependent upon the cognitive domain, but on average is 1.5 standard deviations lower than healthy controls (Saykin et al., 1994). The pathology in the computer model leads to a similar deficit between a “healthy environment” and the schizophrenia condition.

### Implementation of pharmacology for cognitive enhancers

Donepezil is an AChE-inhibitor with a K_*i*_ of 20 nM while galantamine inhibits AChE-I with a much lower affinity of 800 nM and in addition weakly and allosteric potentiates α_7_ and α_4_β_2_ nAChR (Woodruff-Pak et al., 2002). Imaging studies with ^11^C-PMP have suggested that 10 mg donepezil and 24 mg galantamine lead to brain AChE-inhibition levels of 35% (Shinotoh et al., 2001; Darreh-Shori et al., 2008). These clinically observed inhibition levels can be used to calculate the daily dose to affect 50% brain AChE-inhibition, which corresponds to 18.5 mg for donepezil and 44.5 mg for galantamine, resulting in inhibition levels of 20% for 5 mg donepezil, 15% for 8 mg galantamine and 24% for 16 mg galantamine. ACh half-life, T, in the cholinergic receptor competition model is then calculated as T_0_/(1-Enzyme inhibition), with T_0_ being the half-life in untreated patients. The AchE is one of the fastest enzymes in the human body (Iwanaga et al., 1994), leading to a half-life in the untreated situation of 5 ms. This leads to ACh half-lives of 6.9 and 7.7 ms for donepezil at 5 and 10 mg and to half-lives of 5.9, 6.8, and 7.7 ms for galantamine at 8, 16, and 24 mg.

In addition, galantamine has a small allosteric potentiating effect on nAChR (Woodruff-Pak et al., 2002), which we implemented as a 5, 10, or 15% (respectively for 8, 16, and 24 mg) relative increase in both α_7_ nAChR and α_4_β_2_ nAChR activation levels.

### Implementation of smoking

As a disproportionally large fraction of schizophrenia patients smoke (Dalack et al., 1998), we implement the effect of nicotine on both α_4_β_2_ nAChR and α_7_ nAChR. Nicotine has a much higher affinity for α_4_β_2_ nAChR than for a7 nAChR and imaging studies with the PET radiotracer 18F-2-Fluoro-A85380 showed an almost complete saturation of α_4_β_2_ nAChR in smokers (Brody et al., 2006). We assume an increase in α_4_β_2_ nAChR activation of 20% as the receptors are already naturally active. However, this level of α_4_β_2_ nAChR activation, together with the continuous nicotine exposure likely overall leads to receptor desensitization (Grady et al., 2012). Because α_4_β_2_ nAChR regulates GABA release (McClure-Begley et al., 2009; Zappettini et al., 2011) we implement the desenitization induced by the smoking condition as a two-fold decrease in GABA conductances, leading to a greater firing of the network. Given the relative much lower affinity of nicotine for the α_7_ nAChR (20,000 nM vs. 100 nM) (Buisson et al., 1996), we assume smoking does not affect α_7_ nAChR. Note that the amount of ACh bound to α_4_β_2_ (and of α_7_) nAChR is further determined by the galantamine or donepezil mediated AChE inhibition in addition to inhibition of the presynaptic M2 mAchR autoreceptor by specific antipsychotics, such as olanzapine. In our model, this is illustrated by the fact that binding of ACh to α_4_β_2_ nAChR ranges from 24% (non-smoking patient on haloperidol) to 62% (olanzapine in smoking patients on 24 mg galantamine).

### Implementation of memantine pharmacology

Memantine is a relatively weak NMDA-R inhibitor that has a larger affinity for the NMDA-NR2C/2D subunit (Kotermanski and Johnson, 2009) in physiological conditions. Based upon the observation that the NR2C/2D subunits are preferentially located on inhibitory interneurons (Monyer et al., 1994) in rats, memantine's pharmacology is implemented using a two-fold greater inhibition of the NMDAR on interneurons as compared to the NMDAR on pyramidal cells.

Data further suggest that the functional memantine concentration in the human brain is relatively small; in our earlier paper on the cognitive model (Roberts et al., 2012) for Alzheimer's Disease, a 1% decrease in gNMDA on interneurons resulted in a selective positive impact on moderate to severe AD cases but not in the situation of mild cases, suggesting that such an inhibition corresponded to the clinical dose of 20 mg.

### Implementation of pharmacological profile of antipsychotics

**Figure 1:**
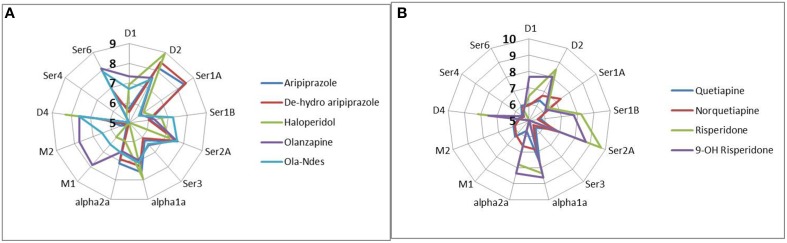
**Complex pharmacology of antipsychotics and their major metabolites (forming the active moiety of currently used antipsychotic medication) illustrated by the pKa (−10logK_i_) against the human receptors, where Ki is the affinity of the drug determined by tracer displacement studies**. More peripheral readouts correspond to higher affinities. Data are derived from the standardized PDSP database (http://pdsp.med.unc.edu/indexR.html). It is clear that many antipsychotics have complex pharmacologies leading to non-linear interactions in the human networks. **(A)** pharmacology of active moiety of aripiprazole, haldoperidol, and olanzapine, **(B)** pharmacology of risperidone and quetiapine.

The affinity parameters for each individual drug and neurotransmitter for human receptors were derived from the standardized PDSP database (http://pdsp.med.unc.edu/indexR.html) (Besnard et al., 2012). Importantly, the active moiety of antipsychotics, taking into account the pharmacology of metabolites was used (see Figure 1). We took great care in determining the functional intrasynaptic concentration of the various antipsychotics using published ^11^C-raclopride displacements observed with specific antipsychotic dose combinations using the receptor competition model, described above.

## Results

### Augmentation therapy with memantine

**Figure 2:**
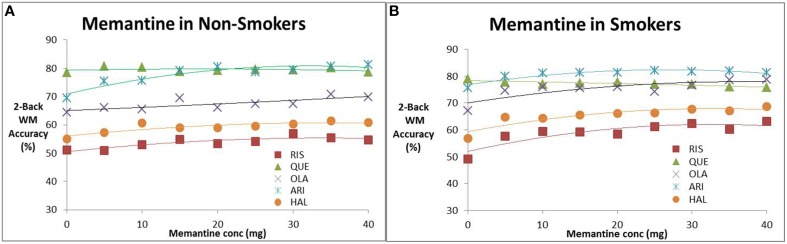
**Effect of increasing memantine dose on anticipated N-back working memory tests outcome (% correct responses in a 2-Back working memory test) in the presence of the five antipsychotics**. **(A)** Non-smokers, **(B)** smokers. The simulations suggest evidence for a dose-dependent effect of memantine for all antipsychotics except quetiapine. The effect is slightly amplified for patients on nicotine. Note that the maximal dose (40 mg) is twice the regular dose used in Alzheimer patients.

We studied the effect of increasing memantine doses on the performance of the *in silico* network for CIAS in the presence of antipsychotics. Figures 2A,B shows the effect of memantine on the estimated 2-back working memory outcomes, respectively in the absence and presence of nicotine. In the absence of smoking, with the exception of quetiapine, all drugs improve cognitive readout with increasing memantine doses with the greatest effect observed for aripiprazole (from 69 accuracy to 81% in a 2-back test). For smoking conditions, the increase in frequency of accurate responses as a function of the optimal memantine dose is amplified for Risperidone (maximal increase from 5 to 14%), Haloperidol (maximal effect increases from 5 to 12%) and olanzapine (maximal effect from 6 to 11%), but not for aripiprazole and quetiapine. Note that this maximal effect happens at memantine doses of 40 mg. At clinically relevant doses of 20 mg, the effect is about half as much.

### Augmentation therapy with AChE-I

**Figure 3:**
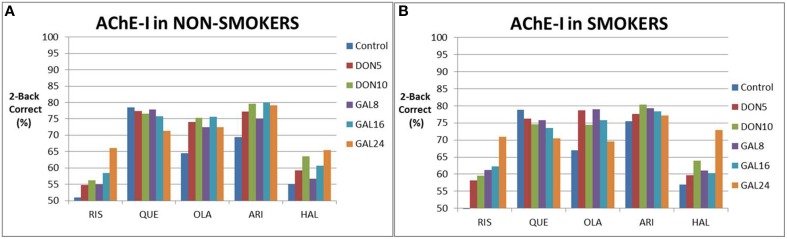
**Simulated clinical outcome (% correct responses in a 2-Back working memory test) for the combination of various cholinomimetics (donepezil and galantamine) with different antipsychotics in non-smoking schizophrenia patients (A) and in smoking schizophrenia patients (B)**. Both galantamine and donepezil show a dose-dependent improvement in the presence of risperidone, aripiprazole, and haloperidol, although the effect sizes differ. However, there is no improvement for quetiapine, probably due to the high baseline, and a more complex dose-response for olanzapine. Smoking tends to slightly enhance the responses of cognition in augmentation therapy with risperidone and haloperidol. However, smoking also tends to slightly decrease the responses of cognition in augmentation therapy with quetiapine and olanzapine. This is probably due to the many non-linear interactions between cholinergic modulation and the complex pharmacodynamics of antipsychotics.

We then simulated the effect of augmentation strategy with ACh inhibitors added to antipsychotics on cognitive outcome (Figure 3), both in the absence and presence of nicotine. In non-smokers, AChE-I dose-dependently improved cognitive outcomes with risperidone, aripiprazole (donepezil only) and haloperidol, with only the 24 mg galantamine showing a robust improvement of >10% in correct responses. In the presence of olanzapine and aripiprazole with galantamine, a tendency was observed for an inverse U-shape dose-response. In the presence of quetiapine, increased AChE-I worsened responses.

Smoking tended to increase the procognitive effect as a stand alone (i.e., without cholinomimetics) and in the presence of risperidone, haloperidol and aripiprazole (donepezil only). For instance the fraction of correct responses went up from 66 to 71% for risperidone with 24 mg galantamine. However, smoking also tended to diminish the cognitive response when augmented with quetiapine and olanzapine. When aripiprazole is augmented with galantamine, smoking shifted the peak response of the inverse U-shaped curve.

### Augmentation therapy with a combination of memantine and AChE-I

This section deals with the simulation of combination therapy of memantine with cholinomimetics on cognitive outcomes. Note that we simulate the outcome of up to five agents in the same patient, i.e., the parent molecule and active metabolite of an antipsychotic, an AChE-I such as donepezil and galantamine, memantine and nicotine. This is a situation that is often encountered in clinical practice.

From the data a complex picture emerges, ranging from a negative effect (adding AChE-I lowers the effect of memantine) with quetiapine and aripiprazole, to an additive effect with risperidone (see Table 1). Synergism is clearly observed with olanzapine and in some cases with haloperidol in the non-smoking case and with haloperidol in the smoking condition.

**Table 1:** **Slopes of the memantine dose-response for non-smokers and smokers, calculated from the trendline of the dose-responses in the different conditions and comparing the effect of adding 5 and 10 mg donepezil and 8, 16, and 24 mg galantamine to memantine in the presence of five different antipsychotics (RIS, risperidone; QUE, quetiapine; OLA, olanzapine; ARI, aripiprazole, HAL, haloperidol)**.

	**Stand-alone**	**DON5**	**DON10**	**GAL8**	**GAL16**	**GAL24**
MEM	0.13	0.16	0.08	0.18	0.07	0.02
RIS NoSmok	0.10	0.05	0.11	0.09	0.10	0.14
QUE NoSmok	0.06	− 0.01	−0.05	0.00	0.00	−0.01
QUE400 NoSmok	0.09	0.03	− 0.03	0.01	−0.07	0.06
QUE600 NoSmok	0.10	0.03	−0.01	0.01	−0.07	0.09
OLA NoSmok	0.08	0.23	0.29	0.09	0.21	0.07
ARI NoSmok	0.23	0.09	0.13	0.12	0.04	−0.01
HAL NoSmok	0.12	0.16	0.18	0.16	0.21	0.16
MEM-SMOK	0.13	0.12	− 0.01	0.13	0.23	0.12
RIS SMOK	0.09	0.04	0.07	0.07	0.08	0.18
QUE SMOK	0.02	0.00	−0.02	−0.01	−0.03	−0.03
QUE400 SMOK	0.05	0.00	− 0.03	0.00	− 0.04	−0.04
QUE600 SMOK	0.01	−0.02	−0.02	−0.02	−0.03	−0.02
OLA SMOK	0.02	0.00	−0.02	−0.01	−0.03	−0.03
ARI SMOK	0.11	0.05	0.03	0.05	0.04	−0.03
HAL SMOK	0.21	0.20	0.16	0.12	0.16	0.13

*The cases with synergy are noted in yellow, while the situation where addition of AChE-I to memantine worsens the dose-response is noted in red. The other cases suggest a pure additive effect. From the data a complex picture emerges, ranging from a negative effect (i.e., lower slopes) in the presence of quetiapine and aripiprazole (in both non-smoking and smoking conditions) to an additive effect with olanzapine and quetiapine combined with donepezil in smoking conditions. Synergism is observed with olanzapine in the non-smoking case with a tendency for synergism in haloperidol non-smokers and with haloperidol in the smoking condition*.

**Figure 4:**
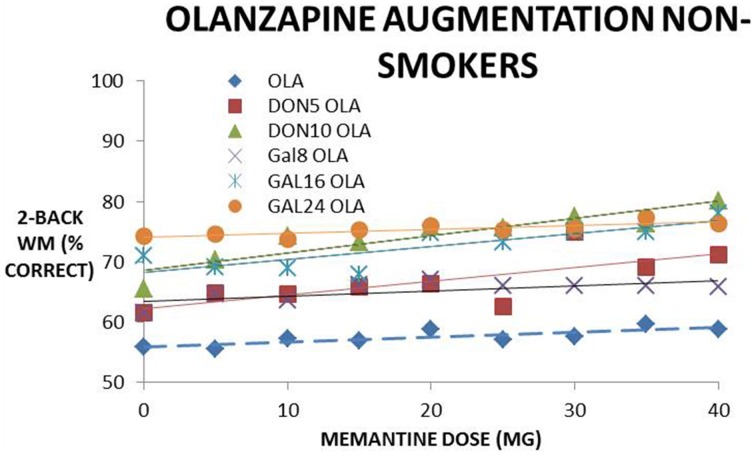
**Simulated clinical outcome (% correct responses in a 2-Back working memory test) for the combination of memantine and cholinomimetics (donepezil and galantamine) as augmentation therapy with olanzapine in non-smoking conditions**. The dose-response of memantine, memantine + donepezil (two doses) and memantine + galantamine (three doses) are fitted with a linear trendline. The slope of the memantine dose-response clearly increases when adding donepezil and galantamine in a dose-dependent way (i.e., with higher donepezil or galantamine doses), suggesting a synergistic effect.

Figure 4 shows a dose-response of memantine on cognitive effects in the presence of olanzapine, and with donepezil or galantamine. Memantine increases cognitive effects dose-dependently in the absence of the AChE-I and the slope (in % correct on the 2-back WM test for 0–40 mg of memantine) of 0.08 is increased to 0.23 and 0.34 when adding donepezil 5 and 10 mg, respectively. When adding galantamine, the slope is increased to 0.13, 0.30, and 0.16 for 8, 16, and 24 mg, respectively. This suggests a synergistic effect with increasing concentrations of donepezil and galantamine, although at the highest galantamine dose of 24 mg the effect is somewhat attenuated. This synergistic effect with olanzapine however disappears in the smoking conditions.

An interesting pattern emerges with regard to different doses of quetiapine. In the non-smoking condition, memantine has a clear beneficial effect on cognition in the absence of any AchE-I with a tendency for greater effect at greater quetiapine doses. Adding donepezil or galantamine reverses this effect at all quetiapine (200–600 mg daily) doses into a negative interaction, i.e., cognitive performance drops with increasing memantine dose. The negative interaction decreases slightly as the quetiapine dose increases. This is likely because adding AchE-I to quetiapine substantially improves cognition; so that further block of the NMDA receptors with memantine reduces GABA tone and drives the network to fire at a very high frequency determined only by the refractory period of the pyramidal neurons, reducing the variability of the interspike distribution and the information bandwidth.

In smoking conditions, the slope of cognitive improvement with memantine without AchE-I is much smaller for all quetiapine doses, because the baseline performance of smoking and quetiapine is already higher than in the non-smoking condition. Adding AchE-I in the smoking condition to quetiapine and memantine has a relative smaller negative effect than in the non-smoking condition, although the absolute values of the slopes are similar. This illustrates the complex non-linear pharmacodynamic interactions between different comedications.

Table 1 shows the slopes of the memantine dose-response with donepezil and galantamine in the presence of specific antipsychotics and smoking/no-smoking conditions. As mentioned above, there is a synergistic effect in non-smokers on olanzapine and in smokers treated with haloperidol for both donepezil and galantamine.

## Discussion

This study addresses the question of a suggested synergistic interaction between AChE-I such as donepezil and galantamine and memantine on cognitive readouts in schizophrenia (Koola et al., 2014) by simulating real-life treatment combinations, including the effect of smoking. We approached this through a mechanism-based QSP computer modeling approach where the pathology of CIAS is combined with the pharmacology and target exposure of the respective drug combinations.

### Combination of AChE-I with antipsychotics

The combination of AChE-I with antipsychotics shows a number of interesting and unexpected outcomes. The 24 mg galantamine has a higher response than any of the two doses of donepezil for risperidone and haloperidol, an effect that is sustained in smokers. Interestingly galantamine shows an inverse U-shape dose-response with olanzapine in non-smokers; the lowest dose of 8 mg had a higher effect than the highest dose. Smoking also drives the responses of both donepezil and galantamine into an inverse dose-response when combined with aripiprazole or olanzapine. One could argue that the interaction of olanzapine with the presynaptic M_2_ mAChR (a Ki of 18 nM) has a disproportionately larger impact given the affinity of ACh for the M_2_ mAChR (which is in the 300 nM range). Blocking the presynaptic autoreceptor could further increase the release of Ach beyond the added effect of AChE inhibition which would then drive the postsynaptic nAChR into desensitization. The level of free ACh in schizophrenia patients is relatively normal, in contrast to the reduced free ACh in Alzheimer's patients, suggesting that the nAChR work on a very different baseline.

Aripiprazole and quetiapine both have strong 5-HT_1_A agonism and lack the inhibition of the presynaptic 5-HT_1_BR that is a hallmark of risperidone; these two properties might account for a relatively good baseline performance as compared to risperidone. The 5-HT_1_A pathway has been documented to play a role in antipsychotic response (Takekita et al., 2015), at least in negative symptoms. Therefore, the higher ACh tone resulting from inhibition of the AChE in the presence of quetiapine and aripiprazole has less dynamical range to improve.

A recent meta-analysis indeed suggest that antipsychotics do indeed have different effects on cognition (Désaméricq et al., 2014), with quetiapine having the greatest beneficial impact on global cognitive score, attention and speed of processing.

The effect of adding AChE-I to antipsychotics on cognition has been studied extensively in clinical trials with effect sizes in the range 0.4–0.6 (Ribeiz et al., 2010; Choi et al., 2013). Studies with donepezil have yielded controversial and mixed results, from positive results (Zhu et al., 2014) to negative results (Kohler et al., 2007; Keefe et al., 2008) with similar negative (Dyer et al., 2008; Lindenmayer and Khan, 2011) and positive results (Schubert et al., 2006; Buchanan et al., 2008) or no effect (Lee et al., 2007) for galantamine leading to the overall perception that these drugs only work marginally or not at all. Our modeling suggests that the nature of the antipsychotic and the condition of smoking does matter. In non-smokers, donepezil and galantamine work best with risperidone, haloperidol, aripiprazole, and olanzapine, but not with quetiapine. In smokers, both galantamine and donepezil enhance cognition with risperidone and haloperidol but have an inverse U-shape dose-response in olanzapine (lower AchE-I doses work best) while there is no effect in aripiprazole and quetiapine. Interestingly high-dose galantamine is inferior to placebo for a number of cognitive readouts in schizophrenia when added to antipsychotics (Dyer et al., 2008). Possible explanations for these observations include the interaction of olanzapine with the muscarinic receptors, especially the presynaptic M_2_ mAChR autoreceptor. This would affect the amount of presynaptically released ACh and therfore interfere in a complex way with the increased half-life of ACh with AChE-I and the allosteric modulatory effect of galantamine. It is of interest to note that the trials with donepezil that showed some efficacy for donepezil, had risperidone and olanzapine as baseline medication (Akhondzadeh et al., 2008; Zhu et al., 2014) with a majority of patients on the lower donepezil dose of 5 mg.

Two trials with galantamine (Schubert et al., 2006; Lindenmayer and Khan, 2011) added to risperidone showed opposite effects. It is worthwhile to examine these two clinical trials in more detail. The positive Schubert trial was a short duration, 8 week trial (*n* = 16), almost all smokers with an average risperidone dose of 5.4 mg (with patients on risperidone for almost 3 years) where anticholinergics were excluded and showed a clear clinical and statistical benefit in delayed memory and attention on the RBANS scale. The negative Lindenmayer trial was a 52-week study of long-acting injection Risperidone (25 and 50 mg) in 32 patients in which galantamine was titrated up to 24 mg; no data on smoking were available. Anticholinergics were allowed for treatment of EPS side-effects, but no data are available on the frequency of this comedication.

Possible clinical trial design differences in these two studies leading to the opposite outcomes include the fraction of smokers (smoking tends to amplify galantamine's effect on cognition) and the somewhat lower dose of 25 mg Ris Consta in the Lindenmayer study as compared to 6 mg oral risperidone. Sensitivity analyses in the model show that lower risperidone doses tend to perform better in the cognitive model, leaving somewhat less dynamic range for additional effect of pro-cognitive enhancers; this is partially explained by the anti-cognitive effect of the dose-dependent presynaptic 5-HT_1_B autoreceptor block that affects free 5-HT levels. Attention deficit has indeed been shown to be correlated with the amount of D_2_R blockade by risperidone (Uchida et al., 2009), at least in late-life schizophrenia; therefore higher doses of risperidone leading to a lower baseline provide a greater dynamic range for pro-cognitive effects of galantamine.

In general, however, the information available from peer-reviewed articles usually does not have the granularity needed to identify the different comedications for individual patients in the different treatment arms. Exploration of other databases such as ADNI, where the comedications are given at the level of individual subjects or the database from electronic health records from the South London and Maudsley National Health Systems registry (Kadra et al., 2015) are a possibility to test the QSP model outcomes to real-life situations.

### Combination of memantine with antipsychotics

The simulations in this paper also suggest that memantine as augmentation therapy has a very modest effect on cognition that is enhanced in the smoking conditions. Again the best response is observed when adding memantine to risperidone and haloperidol, but at concentrations that are about twice as high as currently used in Alzheimer's disease. A meta-analysis of clinical trials (Kishi and Iwata, 2013) indeed suggests a modest effect of memantine on cognition in schizophrenia.

It is worthwhile to expand upon the unexpected clinical pro-cognitive finding of a (weak) NMDA-antagonist. Memantine's interaction under *in vivo* conditions with Mg present, shows a higher affinity for the NMDA-NR2C subunit on the excitatory-inhibitory glutamatergic synapses as compared to the NMDA-NR2A/B subunits that are present on excitatory-excitatory synapses (Kotermanski et al., 2009). Smoking tends to desensitize the α_4_β_2_ nAChR more than the α_7_ nAChR; this would lead to a lower GABA tone as one of α_4_β_2_ nAChR mediated processes regulates the GABA release (McClure-Begley et al., 2009; Zappettini et al., 2011). Memantine, through its antagonism on the excitatory-inhibitory glutamatergic synapse, also lowers the GABA-tone, opening the possibility for an additive or synergistic mechanism by addressing one of the major pathological hallmarks of the schizophrenia condition (Volk and Lewis, 2002).

### Combination of memantine and AChE-I with antipsychotics

When adding AChE-inhibitors to antipsychotics and memantine, the effect greatly depends upon both the nature of the antipsychotic and the smoking condition. Overall, the effect is additive, with the exception of aripiprazole and quetiapine for both smokers and non-smokers and for olanzapine in the smoking condition, where addition of AChE-I decreases the effect of memantine.

A synergistic effect is observed for olanzapine in the non-smoking condition and for haloperidol in both conditions. The observation that adding AChE-I to memantine can improve cognitive readouts is in line with clinical data in Alzheimer's patients (Gauthier and Molinuevo, 2013). The inhibition on the AChE leads to slightly shorter half-life for AChE in the case of galantamine (5.9 ms for 8 mg galantamine vs. 6.5 ms for 5 mg donepezil). The observed lack of differentiation between donepezil and galantamine could be due to the fact that the impact of galantamine's allosteric modulation on nAChR has a quite limited effect (especially given the low efficacy) and is barely capable to compensate for the somewhat lower level of AChE-I. This is in line with clinical observations in the Alzheimer's field that suggest no detectable difference in treatment with either donepezil or galantamine (Tan et al., 2014).

The observation that olanzapine favors a synergistic effect between memantine and AChE-I points to an important role for the GABA interneurons in cortical circuits. Olanzapine has a substantial antagonism at the 5-HT_3_R that regulates GABA interneuron firing (Puig, 2004), a property it shares with clozapine and as such can compensate for the observed GABA dysfunction in schizophrenia (Volk and Lewis, 2002). Indeed, as mentioned above, memantine preferentially interacts with the NMDA receptor subunit on the excitatory-inhibitory synapses, increased levels of ACh through blocking of the AChE can activate α_4_β_2_ nAChR that regulate GABA release and galantamine has an additional allosteric potentiating effect at the same α_4_β_2_ nAChR. The fact that the synergism disappears in smoking olanzapine patients suggests that this interaction is highly non-linear in nature.

Smoking by itself tends to improve cognitive outcome, probably through its effect on α_4_β_2_ nAChR GABA tone, perhaps underscoring its capacity for self-medication. Smoking can also enhance the effect of either memantine or AChE-I in a number of situations, but with both memantine and AChE-I added to antipsychotics the effect is hard to predict due to a number of non-linear interactions. This further underscores the importance of the excitation-inhibition balance in these cortical networks.

It is of interest to elaborate on the negative pharmacodynamic interactions of certain conditions. Increasing evidence suggest that information in the human brain is not encoded in simple firing rates; examples include oscillatory behavior of local field potential in the subthalamic nucleus of basal ganglia that code for motor symptoms (Little et al., 2013; Beudel et al., 2015). Our model readout also takes into account the interspike variability of the action potential train(Geerts et al., 2013) which might explain some of the non-monotonic dose-responses observed with quetiapine and aripiprazole, where increased firing rate does not lead to enhanced cognitive performance.

### Limitations of the model

The network is calibrated on a working memory task, represents only the maintenance phase and therefore probably does not capture the intricacies of the different cognitive tasks. However, we would argue that the network also captures the strength of a memory trace representation that is a necessary step in a number of other cognitive tasks. For instance, in an episodic memory task, an existing memory trace needs to be retrieved from its memory bank and kept for a certain time in memory, although at a different time scale, to perform calculations and to compare it to a novel sensory stimulus.

A major issue is the choice of the biological processes and the changes associated with the pharmacological treatment. For instance, the allosteric effect of galantamine on nAChR has been documented to be as high as 70% (Samochocki et al., 2000) but a later study puts the effect more at 40% and lower (Samochocki et al., 2003). Furthermore, we have somewhat arbitrarily set the effect of smoking as a 20% increase in activation. The effect of such differences can be studied using sensitivity analyses.

Another major limitation is the “blind” pharmacology of antipsychotic drugs; unlike animal models, the computer model is bound by the available knowledge of the pharmacology of the drugs and does not take into account the possible effect of drugs on targets that are undocumented. However, the functional concentration of antipsychotics as measured by PET raclopride radiotracer in clinically relevant situations is in the low nM range, except for quetiapine. This effectively reduces the probability of off-target effects that might play a role at higher doses.

This simulation generates average values for “generic” patients on a fixed dose of an antipsychotic and, memantine and/or an AChE-I. In real-life practice, doses are often titrated to the best trade-off between efficacy and side-effects. In addition, the variability in clinical outcome might be affected by CYTP450 interaction between the different drugs where inhibition or stimulation of a CYTP450 enzyme by one drug might affect the levels of other drugs (so called pharmacokinetic-pharmacokinetic or PK/PK interactions). Finally, variability might be a consequence of different genotypes, for instance the COMTVal158Met and the 5-HTTLPR s/l that all can affect cognitive outcomes. In principle, the QSP platform is able to simulate virtual individual patients complete with PK/PK interactions, different individual doses of the different drugs and any combination of the common genotypes. As an example, we have implemented the COMTVal158Met genotype in the QSP platform using human imaging studies in unmedicated healthy volunteers as a different half-life of dopamine and norepinephrine in cortical synapses (Spiros and Geerts, 2012).

The major message from this simulation exercise is the unexpected impact of different antipsychotics each with their own fingerprint of pharmacological activity on the dose-response of the same drug-drug combination and/or combined with smoking. The non-linear interactions of drug pharmacodynamics plays an important role in real-life drug treatment, as polypharmacy is more the rule than the exception. This is particularly important when considering the design of clinical trials; careful consideration of inclusion/exclusion of comedications can substantially reduce the patient variability in the different treatment arms and increase the probability that a clinical signal can be detected. Failure to take these differential interactions into account might lead to reduction of the clinical signal as treatment arms become inadvertently populated with drugs that do not work or work adversely. We suspect that this might be one of the causes of clinical trial failures of drugs tested with augmentation therapy in Cognitive Impairment with Schizophrenia. Obviously other processes contribute to the variability in clinical trial outcomes such as different genotypes, different pathological baselines and PK variability. In this regard, we would argue that the concept of chlorpromazine equivalents (Beckmann and Laux, 1990) where the differentiation between antipsychotics is solely driven by their dose and their corresponding D_2_R occupancy, although very handy and appealing because of its simplicity, might need a substantial correction.

An additional collorary is that new drugs with a specific selective pharmacology aimed at augmentation therapy could only be combined with specific antipsychotics, because of negative pharmacodynamic interaction of other antipsychotics with specific drug pharmacology of the new investigative compound. In addition, smoking through its effect on nicotinic receptors can significantly affect cognitive outcome. The results of this paper suggest that the complex poly-pharmacy profile of marketed antipsychotics can lead to non-linear pharmacodynamics interactions beyond their simple D_2_R occupancy and that this can significantly impact clinical outcome.

### Conflict of interest statement

The authors declare that the research was conducted in the absence of any commercial or financial relationships that could be construed as a potential conflict of interest.

## Abbreviations

5HTTLPR: Serotonin transporter promotor: long vs. short isoform
ACh: acetylcholine
AChE: acetylcholinesterase
AChE-I: acetylcholinesterase inhibitors
CIAS: Cognitive Impairment in Schizophrenia
COMT: Catechol-O-Methyl Transferase
CYTP450: Cytochrome P450 enzyme
DA: Dopamine
dlPFC: dorso-lateral Prefrontal Cortex
EPS: Extra-pyramidal symptoms
GABA: gamma-amino butyric acid
GPCR: G-protein coupled receptors
mAChR: muscarinic acetylcholine receptor
MATRICS: Measurement And Treatment Research to Improve Cognition in; Schizophrenia
nAChR: nicotinic acetylcholine receptor
NMDA: N-methyl-D-aspartate (glutamate receptor subtype)
PBPD: Physiology-based pharmacodynamic modeling
PBPK: Physiology-based pharmacokinetic modeling
PDSP: Psycho-active Drug Screening Program
PET: Positron Emission Tomography
PFC: Prefrontal Cortex
PK/PK: pharmacokinetic-pharmacokinetic
QSP: Quantitative Systems Pharmacology
RBANS: Repeatable Battery for the Assessment of Neuropsychological Status
R&D: (Pharmaceutical) Research and Development.
